# Clinical significance of subclinical atherosclerosis in retinal vein occlusion

**DOI:** 10.1038/s41598-021-91401-1

**Published:** 2021-06-07

**Authors:** Minhyung Lyu, Yonggu Lee, Byung Sik Kim, Hyun-Jin Kim, Rimkyung Hong, Yong Un Shin, Heeyoon Cho, Jeong-Hun Shin

**Affiliations:** 1 Division of Cardiology, Department of Internal Medicine, Hanyang University Guri Hospital, Hanyang University College of Medicine, 153 Gyeongchun-ro, Guri, Gyeonggi-do 11923 Republic of Korea; 2 Department of Ophthalmology, Hanyang University Guri Hospital, Hanyang University College of Medicine, 153 Gyeongchun-ro, Guri, Gyeonggi-do 11923 Republic of Korea

**Keywords:** Cardiovascular diseases, Eye diseases, Metabolic disorders

## Abstract

Retinal vein occlusion (RVO) is associated with atherosclerotic cardiovascular risk factors; however, its association with the specific markers of subclinical atherosclerosis has not yet been established. To investigate this association, we compared 70 patients with RVO to 70 age- and sex-matched patients without RVO. Low-density lipoprotein cholesterol (LDL-C) levels and brachial-ankle pulse wave velocity (baPWV) were significantly higher in the RVO group than in the control group. Carotid plaques (54.3% vs. 28.6%, p = 0.004) were more frequent in the RVO group. Multivariate logistic regression analysis showed that the presence of carotid plaques (odds ratio [OR]: 3.15, 95% confidence interval [CI] 1.38–7.16, p = 0.006), as well as smoking, LDL-C level, and baPWV were associated with RVO. Additionally, a multinomial logistic regression model showed that the presence of carotid plaques (OR: 3.94, 95% CI 1.65–9.41, p = 0.002) and LDL-C level were associated with branch RVO, whereas smoking and baPWV were associated with central RVO. In conclusion, RVO was associated with subclinical atherosclerosis markers, including carotid plaques and baPWV. These results support the hypothesis that atherosclerosis contributes to the etiology of RVO and suggest the evaluation of subclinical atherosclerosis in patients with RVO.

## Introduction

Retinal vein occlusion (RVO) is the second most common retinal vascular disorder following diabetic retinopathy, and it is a major cause of visual impairment^1^. According to the location of occurrence, RVO is classified into central retinal vein occlusion (CRVO) and branch retinal vein occlusion (BRVO)^1,2^. The pathogenesis of RVO remains largely unknown. Local factors, such as open-angle glaucoma, and systemic conditions, including hypertension, diabetes mellitus, cigarette smoking, and hemostatic abnormalities leading to hypercoagulable states, have been reported as the predisposing conditions in several studies^3–5^.

Patients with RVO have been reported to have an increased risk of cardiovascular disease and cerebrovascular accident^6–9^. Many risk factors for coronary artery disease and stroke, including advanced age, hypertension, dyslipidemia, and diabetes mellitus, are also associated with RVO^10–14^. However, the association of RVO with subclinical atherosclerosis, an early indicator of atherosclerotic burden and overt cardiovascular disease, has not been confirmed. Therefore, we investigated the relationship between RVO and markers of subclinical atherosclerosis, including carotid intima-media thickness (IMT), carotid plaques, and brachial-ankle pulse wave velocity (baPWV), in patients with RVO and those without RVO who had no established cardiovascular diseases.

## Results

**Figure 1:**
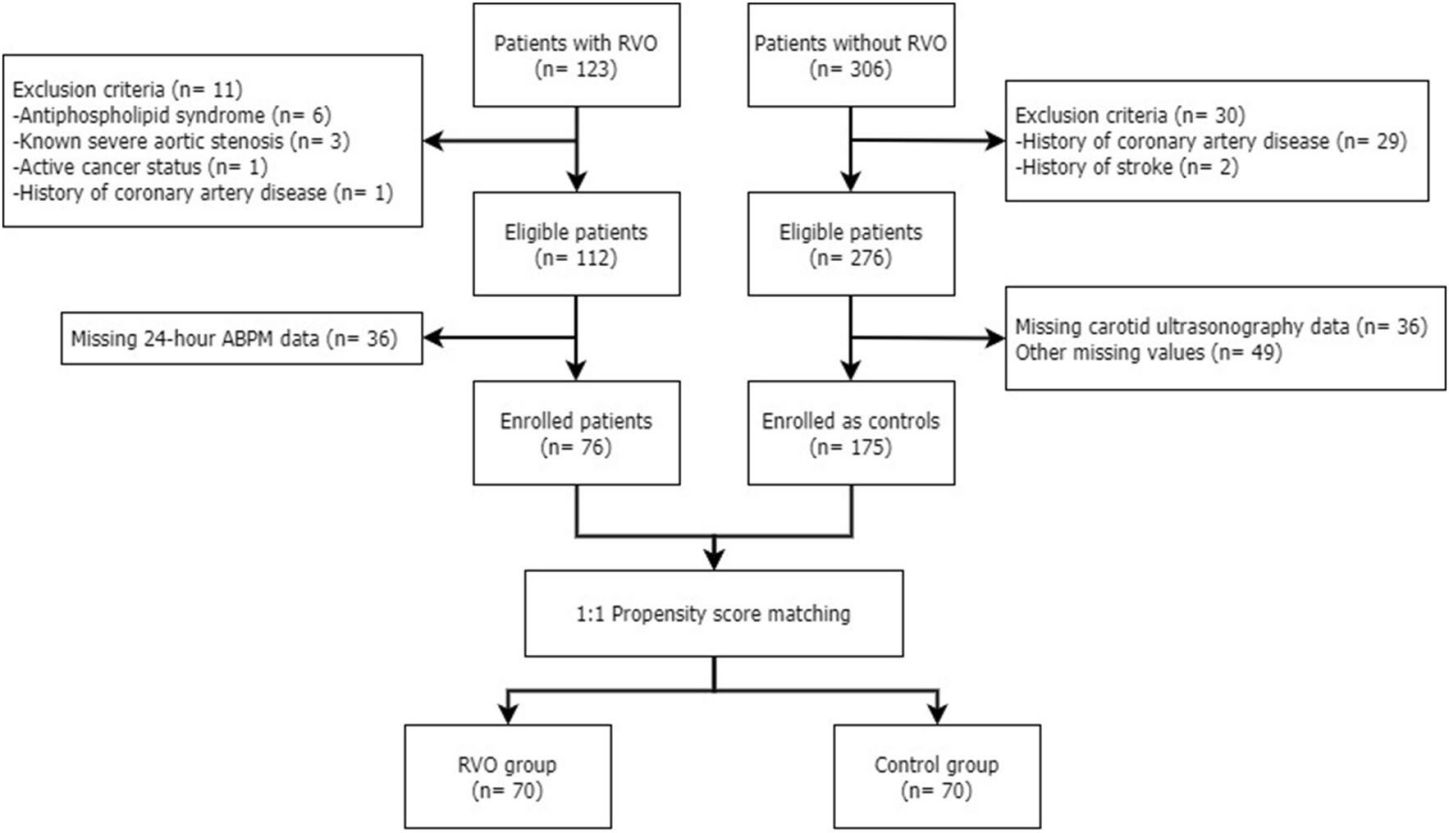
Schematic depiction of the selection process of the study population.

Between January 2015 and February 2019, 76 patients with RVO and 175 control patients, who had no established cardiovascular diseases, were enrolled in a single center. After age and sex were matched using propensity scores, 70 patients with RVO and 70 patients without RVO were finally included in the analysis (Fig. 1). In the unmatched cohort, baseline characteristics showed that patients with RVO were older and slightly more obese, had higher total and low-density lipoprotein (LDL) cholesterol levels, triglyceride levels, and 10-year atherosclerotic cardiovascular disease (ASCVD) risk, and lower estimated glomerular filtration rate (eGFR) levels than those without RVO. In the matched cohort, patients with RVO had higher total and LDL cholesterol levels than those without RVO, but age, body mass index (BMI), and eGFR did not differ between the two groups. D-dimer levels did not differ between the two groups in either the matched or unmatched cohorts (Table 1).

**Table 1:** Baseline characteristics.

	Unmatched cohort				Matched cohort			
	RVO (−)	RVO (+)	*p*-value	SMD	RVO (−)	RVO (+)	*p*-value	SMD
	N = 175	N = 76			N = 70	N = 70		
Age (years)	54.2 ± 15.1	59 ± 10.9	0.016	0.358	58.7 ± 11.4	59 ± 10.9	0.892	0.023
Female sex, n (%)	123 (56.2)	33 (47.1)	0.238	0.181	33 (47.1)	33 (47.1)	0.999	< 0.001
Hypertension, n (%)	169 (77.2)	60 (85.7)	0.172	0.221	55 (78.6)	60 (85.7)	0.377	0.187
Diabetes mellitus, n (%)	27 (12.3)	12 (17.1)	0.409	0.136	13 (18.6)	12 (17.1)	0.999	0.037
Dyslipidemia, n (%)	44 (20.1)	14 (20)	0.999	0.002	12 (17.1)	14 (20)	0.828	0.074
Smoking, n (%)	54 (24.7)	22 (31.4)	0.335	0.151	15 (21.4)	22 (31.4)	0.25	0.228
Current drinking, n (%)	73 (33.3)	27 (38.6)	0.511	0.109	24 (34.3)	27 (38.6)	0.725	0.089
Antiplatelet treatment, n (%)	20 (9.1)	10 (14.3)	0.315	0.161	6 (8.6)	10 (14.3)	0.425	0.18
BMI (kg/m^2^)	24.9 ± 3.6	25.7 ± 3	0.092	0.243	24.9 ± 3.3	25.7 ± 3	0.153	0.243
10-year ASCVD risk (%)	9.3 ± 10.2	12.7 ± 12.1	0.022	0.302	11.1 ± 10.7	12.7 ± 12.1	0.405	0.141
Glucose level (mg/dL)	108 ± 27.7	111.8 ± 29.7	0.325	0.133	114.7 ± 33.8	111.8 ± 29.7	0.59	0.091
HbA1c level (%)	5.7 ± 0.8	5.8 ± 0.9	0.219	0.165	5.9 ± 0.9	5.8 ± 0.9	0.667	0.073
Total cholesterol level (mg/dL)	182.7 ± 36	199.6 ± 40.1	0.001	0.445	183 ± 36.1	199.6 ± 40.1	0.011	0.435
Triglyceride level (mg/dL)	139.2 ± 71.2	156.9 ± 80.1	0.08	0.234	154.1 ± 86.4	156.9 ± 80.1	0.842	0.034
HDL cholesterol level (mg/dL)	53.5 ± 11.8	55.9 ± 19.1	0.208	0.152	52.2 ± 11.2	55.9 ± 19.1	0.158	0.24
LDL cholesterol level (mg/dL)	106.2 ± 24.6	118.2 ± 30	0.001	0.44	107.5 ± 24.9	118.2 ± 30	0.022	0.391
eGFR (mL/min/1.73 m^2^)	98.5 ± 15.4	92.5 ± 15	0.005	0.39	95.7 ± 12.9	92.5 ± 15	0.175	0.231
D-dimer level (ng/mL)	105.7 ± 76.7	106 ± 74.9	0.973	0.005	116.5 ± 96	106 ± 74.9	0.474	0.121
baPWV (cm/s)	1485.7 ± 310.7	1666.4 ± 321.9	< 0.001	0.571	1517.7 ± 291.1	1666.4 ± 321.9	0.005	0.485
ABI	1.11 ± 0.1	1.14 ± 0.07	0.035	0.32	1.11 ± 0.09	1.14 ± 0.07	0.039	0.353
Carotid IMT (mm)	0.64 ± 0.14	0.68 ± 0.12	0.033	0.307	0.67 ± 0.12	0.68 ± 0.12	0.651	0.077
**Carotid plaque, n (%)**								
Unilateral	37 (16.9)	18 (25.7)			16 (22.9)	18 (25.7)		
Bilateral	17 (7.8)	20 (28.6)			4 (5.7)	20 (28.6)		
Total	54 (24.7)	38 (54.3)	< 0.001	0.636	20 (28.6)	38 (54.3)	0.004	0.541

*ABI* ankle-brachial index, *ASCVD* atherosclerotic cardiovascular disease, *baPWV* brachial-ankle pulse wave velocity, *BMI* body mass index, *eGFR* estimated glomerular filtration rate, *HbA1c* hemoglobin A1c, *HDL* high-density lipoprotein, *IMT* intima-media thickness, *LDL* low-density lipoprotein, *RVO* retinal vein occlusion, *SMD* standardized mean difference.

**Figure 2:**
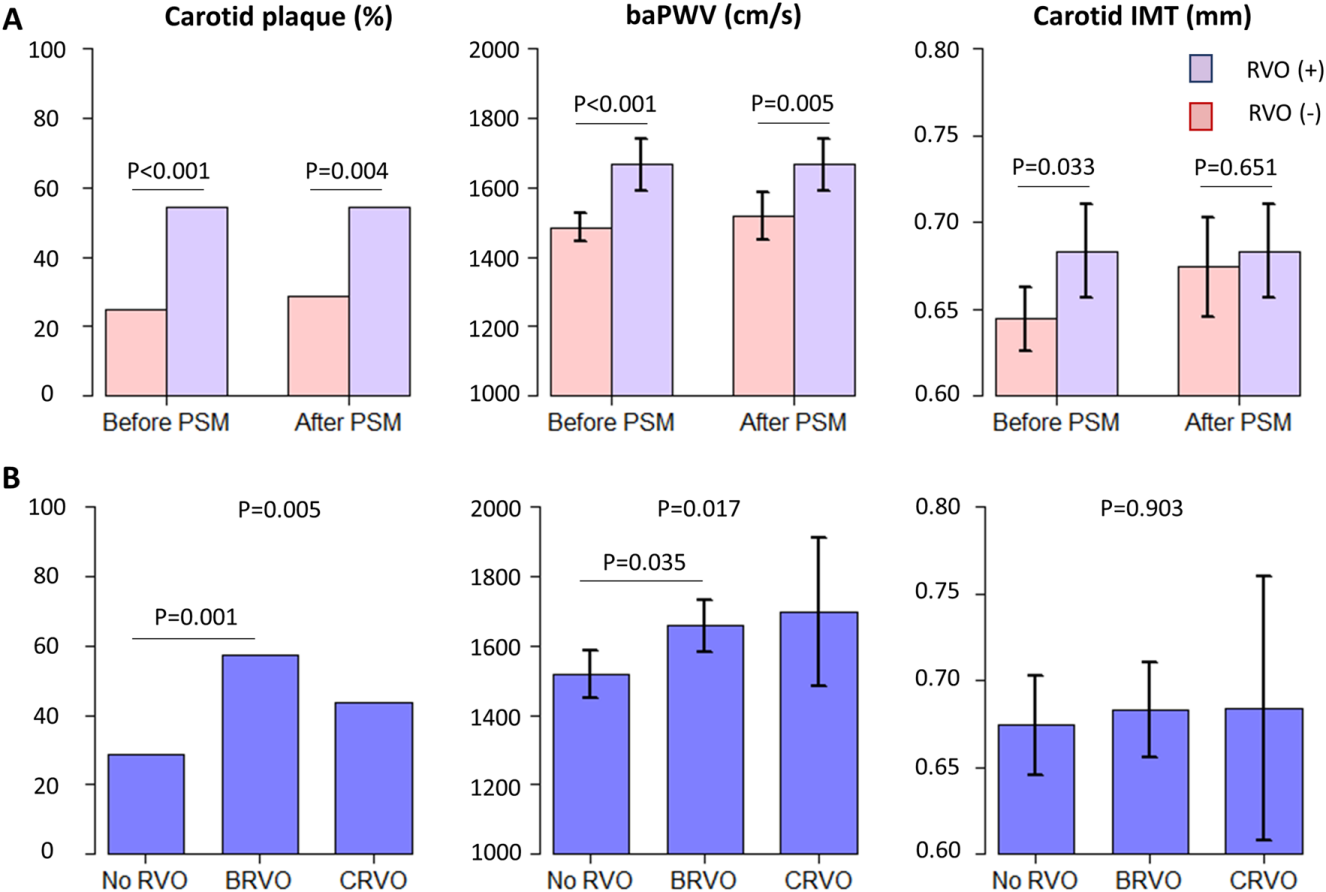
Markers of subclinical atherosclerosis in patients with and without RVO. (**A**) The frequencies of carotid plaque and baPWVs were higher in patients with RVO than in those without RVO in both matched and unmatched cohorts, whereas the carotid IMT was not different between the two groups in the matched cohort. (**B**) In the matched cohort, patients with BRVO had higher frequencies of carotid plaque and higher baPWV levels than those without RVO, whereas the frequencies of carotid plaque and baPWVs were not different between the patients with CRVO and those without RVO. The carotid IMT was not different among the three groups. This figure was created using R with the “graphics” package (R Core Team, 2020)^15^. *baPWV* brachial-ankle pulse wave velocity, *RVO* retinal vein occlusion, *BRVO* branch retinal vein occlusion, *CRVO* central retinal vein occlusion, *IMT* intima-media thickness, *PSM* propensity score matching.

The presence of carotid plaques and baPWV was higher in patients with RVO than in those without RVO in both the matched and unmatched cohorts. In contrast, carotid IMT was not different between the two groups in the matched cohort, while the patients with RVO had higher carotid IMTs than those without RVO in the unmatched cohort (Fig. 2A).

Univariate binary logistic regression models showed that LDL cholesterol levels, baPWV, and the presence of carotid plaques were associated with the occurrence of RVO in the matched cohort. The multivariate binary logistic model showed that ever-smoking, LDL cholesterol level, baPWV, and the presence of carotid plaques were independently associated with the occurrence of RVO, whereas carotid IMT was not in the matched cohort (Table 2).

**Table 2:** Logistic regression analysis for predictors of RVO.

	Univariate			Multivariate		
	OR	95% CI	*p*-value	OR	95% CI	*p*-value
Age (per 10 years)	1.02	0.76–1.38	0.891	–	–	–
Female sex	1.00	0.51–1.94	1.000	–	–	–
Hypertension	1.64	0.68–3.94	0.273	–	–	–
Diabetes mellitus	0.91	0.38–2.16	0.825	–	–	–
BMI ≥ 25 kg/m^2^	1.59	0.81–3.10	0.176	–	–	–
Current drinking	1.20	0.60–2.40	0.598	–	–	–
Smoking	1.68	0.78–3.60	0.182	2.86	1.16–7.04	0.022
LDL cholesterol (per 30 mg/dL)	1.54	1.06–2.24	0.024	1.60	1.05–2.44	0.030
Carotid IMT (per 0.1 mm)	1.07	0.81–1.41	0.648	–	–	–
baPWV (per 5 m/s)	2.25	1.25–4.04	0.007	2.00	1.03–3.89	0.041
**Carotid plaque**						
Total	2.97	1.47–5.98	0.002	3.15	1.38–7.16	0.006
Unilateral	1.76	0.78–3.94	0.170	–	–	–
Bilateral	7.81	2.45–25.0	0.001	–	–	–

*baPWV* brachial-ankle pulse wave velocity, *BMI* body mass index, *CI* confidence interval, *IMT* intima-media thickness, *LDL* low-density lipoprotein, *OR* odds ratio, *RVO* retinal vein occlusion.

Of the 70 patients with RVO, 53 and 16 had BRVO and CRVO, respectively. One patient was classified as either of the RVOs because he was diagnosed with hemi-CRVO. We compared the baseline characteristics of patients with CRVO and BRVO with those of patients without RVO in the matched cohort. Among the three patient groups, diabetes mellitus and dyslipidemia were most prevalent and hemoglobin A1c levels were highest in patients with CRVO, whereas total and LDL cholesterol levels were highest in patients with BRVO. Age, number of males, frequency of ever-smoking, alcohol intake, antiplatelet agent use, BMI, 10-years ASCVD risk, eGFR, and D-dimer levels were not significantly different among the three patient groups (Supplementary Table S1). The presence of carotid plaques and baPWV was higher in patients with BRVO than in those without RVO, whereas there was no significant difference between patients with CRVO and those without RVO (Fig. 2B).

Univariate multinomial logistic regression models showed that LDL cholesterol levels, baPWV, and the presence of carotid plaques were associated with BRVO, whereas ever-smoking and baPWV were associated with CRVO in the matched cohort. The multivariate multinomial logistic regression model showed that the LDL cholesterol level and the presence of carotid plaques were significantly associated with the occurrence of BRVO, whereas ever-smoking was significantly associated and baPWV was marginally associated with the occurrence of CRVO in the matched cohort (Table 3).

**Table 3:** Multinomial regression for predictors of CRVO and BRVO.

	Univariate				Multivariate			
	BRVO group vs. control group		CRVO group vs. control group		BRVO group vs. control group		CRVO group vs. control group	
	OR	*p*-value	OR	*p*-value	OR	*p*-value	OR	*p*-value
Age (per 10 years)	1.00 (0.73–1.38)	0.991	1.09 (0.67–1.78)	0.732	–	–	–	–
Female sex	1.12 (0.55–2.28)	0.752	0.67 (0.22–2.05)	0.486	–	–	–	–
Hypertension	1.36 (0.55–3.41)	0.507	4.09 (0.5–33.52)	0.189	–	–	–	–
Diabetes mellitus	0.55 (0.19–1.55)	0.257	2.63 (0.81–8.54)	0.108	–	–	–	–
BMI ≥ 25 kg/m^2^	1.80 (0.87–3.71)	0.112	1.06 (0.36–3.14)	0.918	–	–	–	–
Current drinking	1.22 (0.58–2.55)	0.597	1.15 (0.37–3.55)	0.808	–	–	–	–
Smoking	1.41 (0.62–3.22)	0.414	2.85 (0.91–8.92)	0.072	2.41 (0.91–6.34)	0.075	4.58 (1.26–16.62)	0.021
LDL cholesterol (per 30 mg/dL)	2.17 (1.39–3.38)	0.001	0.58 (0.30–1.12)	0.103	2.31 (1.42–3.75)	0.001	0.61 (0.31–1.21)	0.156
Carotid IMT (per 0.1 mm)	1.07 (0.79–1.44)	0.679	1.07 (0.68–1.69)	0.760	–	–	–	–
baPWV (per 5 m/s)	2.16 (1.17–4.00)	0.014	2.56 (1.09–6.03)	0.032	1.83 (0.92–3.66)	0.086	2.54 (0.98–6.59)	0.056
Carotid plaque	3.37 (1.59–7.12)	0.001	1.94 (0.64–5.93)	0.243	3.94 (1.65–9.41)	0.002	2.01 (0.54–7.5)	0.300

*baPWV* brachial-ankle pulse wave velocity, *BMI* body mass index, *BRVO* branch retinal vein occlusion, *CI* confidence interval, *CRVO* central retinal vein occlusion, *IMT* intima-media thickness, *LDL* low-density lipoprotein, *OR* odds ratio, *RVO* retinal vein occlusion.

## Discussion

The main findings of this study were as follows: (1) the presence of carotid plaques and baPWV were higher in patients with RVO than in those without RVO; (2) smoking, LDL cholesterol level, baPWV, and the presence of carotid plaques were independently associated with the development of RVO; (3) LDL cholesterol level and the presence of carotid plaques were significantly associated with the development of BRVO, whereas smoking was significantly associated and baPWV was marginally associated with the occurrence of CRVO. To the best of our knowledge, this study is the first to show that the markers for subclinical atherosclerosis, including carotid plaque and baPWV, were associated with the development of RVO.

The pathophysiology of RVO is still unclear, but it is thought that age-related alterations of collagen tissue causing stiffening of the lamina cribrosa and/or atherosclerosis of retinal arteries inducing remodeling and thickening of the arterial wall may cause compression of the adjacent veins within the shared adventitial sheath, leading to blood flow stasis and formation of an endoluminal thrombus^5,16^. Previous studies have reported associations between the presence of RVO and traditional cardiovascular risk factors, such as hypertension, dyslipidemia, diabetes mellitus, and cigarette smoking^16^. Subsequently, a hypothesis had been raised that atherosclerosis may play an important role in the development of RVO; however, there is limited published data demonstrating the associations between the occurrence of RVO and the presence of atherosclerosis.

Atherosclerosis is a chronic inflammatory disease of the arteries, which is the most common pathophysiological process underlying cardiovascular disease. Atherosclerosis exists on a continuum from subclinical atherosclerosis to clinical atherosclerotic cardiovascular diseases including myocardial infarction and stroke. The presence of carotid plaques and increased pulse wave velocity, well-established markers of subclinical atherosclerosis are early indicators of atherosclerotic burden. Timely recognition of their existence would be important to intervene in the progression of subclinical atherosclerosis to overt cardiovascular diseases, and it would be an optimal approach for the primary and secondary prevention of RVO^17^. Our results demonstrate that the presence of carotid plaques and increased baPWV were independently associated with RVO, highlighting the close relationship between retinal microvascular abnormalities and systemic atherosclerosis. Our results also showed that LDL cholesterol levels and the presence of carotid plaques were associated with the presence of BRVO, whereas smoking was associated with the presence of CRVO. These differences in the significant predictors may reflect the pathophysiologic differences between BRVO and CRVO. Because the retinal artery and its corresponding vein share a common adventitial sheath, thickening of the artery appears to compress the vein. This causes secondary changes, including venous endothelial cell loss, thrombus formation, and potential occlusion. BRVO predominantly occurs at arteriovenous crossing sites where the retinal artery may compress the retinal vein to narrow the lumen^18,19^. Hyperlipidemia is a major risk factor for atherosclerosis, and abnormal lipid metabolism is an important component of atherosclerosis. It has been known that LDL is the most abundant atherogenic lipoprotein in plasma and is the main source of cholesterol accumulated within the arterial wall^20^. Consistent evidence from a broad spectrum of clinical and genetic studies has shown a log-linear relationship between the absolute changes in plasma LDL cholesterol levels and the risk of clinical atherosclerotic disease^21–23^. In addition, the association between dyslipidemia, including elevated LDL cholesterol levels, and the development of RVO has been established in previous studies^3,24,25^. Therefore, retinal artery atherosclerosis plays an important role in the pathogenesis of BRVO, and it may correlate with atherosclerosis in the carotid arteries. In contrast, CRVO is associated with thrombus formation resulting from endothelial dysfunction in the retinal veins near the lamina cribrosa, where the retinal veins are normally narrowed, which can be easily promoted by smoking^26,27^. Further studies are needed to determine the exact role of subclinical atherosclerosis in the development of BRVO and CRVO.

In the present study, we documented that the prevalence of carotid plaque was higher in patients with RVO and independently associated with RVO, especially BRVO. Traditionally, carotid plaque and carotid IMT have been used as surrogate markers for atherosclerotic disease. However, there is debate about the better marker and whether carotid plaques have a stronger association with atherosclerotic disease than carotid IMT^28–30^. Data from previous studies have shown an association between RVO and atherosclerosis, although these associations have not been consistent^31,32^. A large population-based cohort study on multiethnic groups reported that the markers of subclinical atherosclerosis, including carotid IMT, coronary artery calcium scores and ABI, were not associated with the presence of RVO, while atherosclerosis risk factors including hypertension and dyslipidemia were^24^. Similarly, Rath et al. also reported that prior coronary artery disease or stroke was not associated with the presence of RVO in a case–control study^33^. In contrast, Matsushima et al. found carotid plaques in 19 of 39 CRVO patients (49%) and in 4 of 18 BRVO patients (22%)^30^, and Martinez et al. found carotid plaques in 36 of 48 (75%) patients with RVO^34^. Although these two studies may have shown substantially high incidences of carotid plaques in patients with RVO, they could not provide evidence for the association between carotid atherosclerosis and the presence of RVO because of the lack of a control group. On the contrary, Wong et al. showed on pooled data from two large population-based cross-sectional cohort studies, Atherosclerosis Risk in Communities Study and the Cardiovascular Health Study, that the presence of carotid plaques increased the risk of RVO (OR: 4.62; 95% CI 1.85–11.6)^31^, similar to our results. In addition, they showed that the risk of RVO was associated with current smoking, which is consistent with our results. Although our study was a relatively small case–control study compared with the study reported by Wong et al., the largest population with RVO among those in published studies, we included 70 patients with RVO and selected a small but balanced control group using the propensity scores. We also compared the differences in the significant predictors between CRVO and BRVO, which has not been reported in the literature. Further studies are needed to clarify the characteristics of carotid plaques in patients with RVO, and their association with pathophysiology, clinical course, complications, and treatment prognosis.

Measurement of arterial stiffness is clinically important because it is associated with a patient’s future cardiovascular events, independent of traditional cardiovascular risk factors^35,36^. We documented that higher baPWV, representing increased arterial stiffness, was associated with the development of RVO, which supports the potential role of atherosclerosis in the pathogenesis of RVO. This result was similar to the findings of studies showing increased arterial stiffness in patients with RVO by Kaderli et al.^37^ and Nakazato et al.^38^. However, these studies were conducted exclusively in patients with BRVO and did not control for confounding variables in statistical analysis. Our results are consistent with those of a previous study by Gouliopoulos et al.^39^, which stated that patients with RVO have increased arterial stiffness by measuring the carotid-femoral pulse wave velocity, and that elevated pulse wave velocity is significantly associated with RVO. However, their study had a limitation in that they had a relatively small sample size and did not show the results according to the classification of RVO (BRVO or CRVO). Our study was conducted with a larger number of patients and minimized the impact of multiple confounders by including a variety of variables in the analysis. Furthermore, we suggest that the role of arterial stiffness is greater in patients with CRVO than in those with BRVO.

This study had several limitations. First, it was a single-center observational case–control study; therefore, any associations observed in the results cannot directly be interpreted as causality, and there may be institutional biases lurking in the data. Second, the sample size was small, especially for the number of patients with CRVO, which represents the scarcity of RVO in the real world and the even lower incidence of CRVO than that of BRVO. The small sample size may raise issues related to statistical power in the analysis results and overfitting of regression models. Regarding overfitting, both the binary and multinomial multivariate models were reduced using the respective variable selection methods to the models with four significant predictors. These procedures minimize the potential overfitting biases. We also provided the statistical power for our results (Supplementary Tables S2 and S3). The multivariate binary logistic model was slightly underpowered for ever-smoking (0.714) and LDL cholesterol levels (0.776), therefore, no definite conclusions can be drawn from the association of ever-smoking and high LDL-C levels with RVO. However, the model was sufficiently powered for baPWV (0.981) and the presence of carotid plaques (0.865) which supports our primary results. The multivariate multinomial model was sufficiently powered, not only for the significant predictors of BRVO, but also for those of CRVO (0.801 for smoking and 0.900 for baPWV). Third, the control subjects without RVO had more cardiovascular risk factors than the general population because they were recruited from the cardiology outpatient clinic. To minimize the differences in patient characteristics, we excluded patients with overt cardiovascular diseases and matched age and sex between the groups using propensity scores. Finally, we did not investigate the type of treatment and cardiovascular/ophthalmologic outcomes after RVO. Further larger scale, longitudinal cohort studies are required to investigate long-term cardiovascular outcomes related to RVO.

As discussed above, the role of atherosclerosis in RVO development is crucial. Surveillance for the presence of subclinical atherosclerosis can help in early risk management and improve health outcomes of atherosclerosis-related diseases. However, there is no established tool for evaluating subclinical atherosclerosis in patients with RVO. Our study suggests that carotid ultrasound and baPWV may be useful tools to evaluate atherosclerosis and aid in preventing complications such as coronary artery disease and stroke in patients with RVO. In this regard, further studies are needed to evaluate the effectiveness of surveillance for subclinical atherosclerosis using carotid ultrasonography and baPWV for improvement in the prevention and treatment of RVO.

In conclusion, we found that RVO was significantly associated with the presence of subclinical atherosclerosis, represented by carotid atherosclerotic plaques and increased baPWV. Our results highlight the potential role of atherosclerosis in the pathogenesis of RVO. Assessing subclinical atherosclerosis using carotid ultrasonography and baPWV measurement may be useful to evaluate cardiovascular risk and provide tailored management targeting long-term clinical outcomes in patients with RVO.

## Methods

### Study design

A prospective, case–control study was conducted at a tertiary referral center. Patients diagnosed with RVO in the center between January 2015 and February 2019 were consecutively enrolled in this study. Patients without established ophthalmologic diseases who visited the outpatient cardiology clinic for cardiovascular disease screening evaluation were enrolled in the control group. Both patients with RVO (RVO group) and those from the control group underwent screening evaluations for clinical cardiovascular diseases through detailed history taken by a cardiologist, laboratory tests, chest radiography, and electrocardiography, and underwent work-ups for subclinical atherosclerosis, including 24-h ambulatory blood pressure monitoring (ABPM), carotid ultrasonography, and baPWV measurements. Patients with the following criteria were excluded from both groups: (1) RVO diagnosed more than 12 months prior to enrollment; (2) the presence of any established atherosclerotic cardiovascular diseases, including coronary artery disease and stroke; and (3) the presence of heart failure, malignancy, liver cirrhosis, end-stage renal disease, and systemic autoimmune disease. Written informed consent was obtained from all patients for reviewing their medical records. The study was approved by the Institutional Review Board of Hanyang University and conducted in accordance with the Declaration of Helsinki.

### Ophthalmological examination

A comprehensive ophthalmic examination, including best-corrected visual acuity, refractive errors, intraocular pressure, biomicroscopy, and fundoscopy, was conducted in all patients with RVO. Swept-source optical coherence tomography (DRI OCT Triton, Topcon Corporation, Tokyo, Japan), ultra-wide fundus photography, and fluorescein angiography (Optos California; Optos PLC, Dunfermline, United Kingdom) were used to confirm the diagnosis and determine the degree of retinal ischemia and the presence of macular edema. RVO diagnosis was determined by retinal specialists. Patients with other concomitant ocular diseases (diabetic retinopathy, age-related macular degeneration, uveitis, epiretinal membrane, macular hole in either eye), history of ocular trauma or vitreoretinal surgery, low-quality OCT or fundus images, and high refractive errors (spherical equivalent >  ± 6) were excluded.

### Clinical and laboratory evaluation

Demographic and clinical characteristics, including age, sex, smoking status, and comorbidities, such as hypertension, diabetes mellitus, and dyslipidemia, were obtained through a review of medical records. Laboratory test results for lipid profiles, blood glucose levels, hemoglobin A1c levels, eGFR, and D-dimer levels were also collected. Hypertension was defined as the use of antihypertensive medications or an average systolic blood pressure ≥ 130 mmHg and/or an average diastolic blood pressure ≥ 80 mmHg in the 24-h ABPM^40^. Diabetes mellitus was defined as the use of oral hypoglycemic agents or insulin, or a hemoglobin A1c level ≥ 6.5%. The 10-year ASCVD risk was estimated using age, sex, smoking status, total cholesterol level, high-density lipoprotein cholesterol (HDL-C) level, systolic blood pressure, and treatment status for hypertension and diabetes mellitus^41^.

### Assessment of subclinical atherosclerosis

The carotid arteries were assessed using an ultrasound system (IE33; Philips Healthcare, Andover, MA, USA) equipped with an 11 MHz linear array probe. An experienced diagnostic medical sonographer performed carotid IMT measurements, using a semi-automated edge-detection software, and calculated the mean carotid IMT value from both common carotid arteries at the end-diastole in a 10-mm segment located 10 mm proximal to the carotid bulb. The mean carotid IMT of both carotid arteries was used in this study. Carotid plaque was defined as focal thickening of carotid IMT > 15 mm or > 50% of the surrounding wall^42^. To evaluate arterial stiffness non-invasively, baPWV and ABI were measured using an oscillometric sphygmomanometric device (VP-1000 plus; Omron Colin, Kyoto, Japan)^43^. The procedure was performed with the patient in the supine position after a 5-min rest. Cuffs were applied to both the brachia and ankles. Blood pressure, pulse volume waveform, and heart rate were simultaneously measured. ABI was defined as the ratio of the systolic blood pressure at the ankle to the systolic blood pressure from either arm (whichever being the highest). The mean values of the left and right baPWVs and ABI were used in the analysis.

### Propensity score matching procedures and statistical analyses

Initially, 123 patients were included in the RVO group, and 306 patients were included in the control group. In the RVO group, six patients with anti-phospholipid syndrome, three patients with severe aortic valve stenosis, one patient with active cancer, and one patient who underwent percutaneous coronary intervention due to unstable angina were excluded. In addition, 36 patients with inadequate 24-h ABPM data were excluded. After excluding patients satisfying any of the exclusion criteria and those with missing values in their records, 76 patients in the RVO group and 175 patients in the control group remained for the final statistical analysis (Fig. 1).

To reduce the differences in demographics between the two groups caused by the discrepancy in enrollment during the comparisons of the subclinical atherosclerosis markers, age and sex were balanced between the groups using a propensity score matching procedure. The matching procedure was conducted in a 1:1 ratio using the nearest neighbor method. The quality of the matching procedure was assessed using absolute mean differences (Supplementary Fig. S1).

The categorical variables were described as numbers (%) and were compared using the Chi-squared test. Continuous variables were described as mean ± standard deviation (SD) and were compared using the Student’s t-test. Univariate binary logistic regression analyses were performed to evaluate the association between clinical factors and the presence of RVO. Multivariate binary logistic regression analyses were used to determine whether an independent association between subclinical atherosclerosis markers and RVO was evident in the presence of confounding factors. The covariates of the multivariate binary logistic models included age, sex, hypertension, diabetes mellitus, BMI, antiplatelet agent use, alcohol intake, smoking, LDL cholesterol level, baPWV, carotid IMT, and the presence of carotid plaques. The model was reduced using a backward selection method (cut-off criterion p > 0.05) to avoid overfitting and to identify the strong predictors of RVO.

We also conducted another set of analyses consisting of comparisons among the three groups of patients in the matched cohort: patients with CRVO, with BRVO, and without RVO. Post-hoc analysis for ANOVA was conducted using a TukeyHSD test. Categorical variables were compared among the groups using the Chi-squared test, and continuous variables were compared using analysis of variance. Multinomial logistic regression analyses were performed to compare the differences in the impact of subclinical atherosclerosis markers on the presence of CRVO and BRVO. The same list of variables used in the binary logistic models was also employed as a covariate in the multinomial logistic regression analyses. The multinomial model was also reduced through a backward variable selection process using the Akaike information criterion (AIC). The best-fit model was selected at the lowest AIC level. All statistical analyses were performed using the R statistical software (ver. 4.0; R Foundation for Statistical Computing, Vienna, Austria) and RStudio (ver. 1.3; RStudio Inc., Boston, MA, USA) and their packages, including “rms”, “matchIt”, “descry”, and “tableone”. Statistical powers were calculated for the binary logistic regression models and multinomial logistic regression models using commercially available statistical software PASS 2008. Statistical significance was set at p ≤ 0.05.

## Supplementary Information

Supplementary Information.

## Data Availability

The datasets generated and/or analyzed during the current study are available from the corresponding author on reasonable request.
