# Supplementary material for: Clinical significance of subclinical atherosclerosis in retinal vein occlusion
Source: Sci Rep. 2021 Jun 7;11:11905. doi: 10.1038/s41598-021-91401-1 (PMC8184809; doi:10.1038/s41598-021-91401-1)
Supplement: Supplementary file 1 — Supplementary Information. [file 41598_2021_91401_MOESM1_ESM.pdf]

Supplementary materials

Clinical significance of subclinical atherosclerosis in retinal vein occlusion

Minhyung Lyu[1†]; Yonggu Lee[1†]; Byung Sik Kim[1]; Hyun-Jin Kim[1]; Rimkyung Hong, MD[2]; Yong Un Shin[2*]; Heeyoon Cho[2]; Jeong-Hun Shin[1*]

[1] Division of Cardiology, Department of Internal Medicine, Hanyang University Guri Hospital, Hanyang University College of Medicine, Guri, Republic of Korea

[2] Department of Ophthalmology, Hanyang University Guri Hospital, Hanyang UniversityCollege of Medicine, Guri, Republic of Korea

[†] These authors contributed equally to this work.

[*] **Co-corresponding authors:**

Yong Un Shin, M.D., Ph.D., Department of Ophthalmology, Hanyang University Guri Hospital, Hanyang University College of Medicine, 153 Gyeongchun-ro, Guri, Gyeonggi-do 11923, Republic of Korea; Phone: +82-31-560-2168; Fax: +82-31-564-9479; E-mail: yushin@hanyang.ac.kr

Jeong-Hun Shin, M.D., Ph.D., Division of Cardiology, Department of Internal Medicine, Hanyang University Guri Hospital, Hanyang University College of Medicine, 153 Gyeongchun-ro, Guri, Gyeonggi-do 11923, Republic of Korea; Phone: +82-31-560-2216; Fax: +82-31-560-2219; E-mail: cardio.hyapex@gmail.com

Supplementary Table S1. Baseline characteristics according to the type of RVO in the matched cohort

	Control	BRVO	CRVO			Post-hoc analysis		
	N=70	N=53	N=16	p-value	SMD	Control vs. BRVO	Control vs. CRVO	BRVO vs. CRVO
Age (years)	58.7 ± 11.4	58.7 ± 9.8	59.8 ± 14.3	0.941	0.056	1.000	0.939	0.944
Female sex, n (%)	33 (47.1)	27 (50)	6 (37.5)	0.679	0.169	0.893	0.674	0.552
Hypertension, n (%)	55 (78.6)	45 (83.3)	15 (93.8)	0.345	0.301	0.663	0.293	0.523
Diabetes mellitus, n (%)	13 (18.6)	6 (11.1)	6 (37.5)	0.052	0.43	0.372	0.189	0.037
Dyslipidemia, n (%)	12 (17.1)	7 (13)	7 (43.8)	0.019	0.483	0.697	0.048	0.019
Smoking, n (%)	15 (21.4)	15 (27.8)	7 (43.8)	0.181	0.325	0.544	0.126	0.367
Current drinking, n (%)	24 (34.3)	21 (38.9)	6 (37.5)	0.866	0.064	0.734	1.000	1.000
Antiplatelet medication, n (%)	6 (8.6)	6 (11.1)	4 (25)	0.175	0.301	0.867	0.156	0.323
BMI (kg/m^2)	24.9 ± 3.3	25.7 ± 3	25.7 ± 3.3	0.361	0.162	0.381	0.648	1.000
10-year ASCVD risk (%)	11.1 ± 10.7	11.3 ± 10.6	17.2 ± 15.7	0.134	0.309	0.991	0.124	0.162
Glucose level (mg/dL)	114.7 ± 33.8	109.9 ± 27.5	118.3 ± 36.5	0.562	0.174	0.680	0.912	0.622
HbA1c level (%)	5.9 ± 0.9	5.7 ± 0.6	6.4 ± 1.4	0.009	0.502	0.280	0.077	0.006
Total cholesterol level (mg/dL)	183 ± 36.1	207.6 ± 35.9	172.8 ± 42.7	<0.001	0.608	0.001	0.573	0.003
Triglyceride level (mg/dL)	154.1 ± 86.4	158 ± 80.9	153.1 ± 79.5	0.96	0.04	0.964	0.999	0.977
HDL level (mg/dL)	52.2 ± 11.2	56.2 ± 18.2	55 ± 22.7	0.357	0.161	0.335	0.791	0.962
LDL level (mg/dL)	107.5 ± 24.9	125 ± 26.9	95.4 ± 29.3	<0.001	0.724	0.001	0.225	0.000
eGFR (mL/min/1.73 m^2)	95.7 ± 12.9	92.6 ± 15.1	92.2 ± 14.9	0.398	0.168	0.438	0.633	0.994
D-dimer level (ng/mL)	116.5 ± 96	99.5 ± 72.5	128 ± 80.8	0.394	0.234	0.522	0.879	0.476
baPWV (cm/s)	1517.7 ± 291.1	1657.6 ± 283.4	1696.1 ± 437.6	0.017	0.357	0.035	0.095	0.899
ABI	1.11 ± 0.09	1.14 ± 0.06	1.14 ± 0.08	0.116	0.261	0.153	0.325	0.975
Carotid IMT (mm)	0.67 ± 0.12	0.68 ± 0.1	0.68 ± 0.16	0.903	0.053	0.912	0.951	0.999
Carotid plaque								
None	50 (71.4)	23 (42.6)	9 (56.2)	0.003	0.527	<0.001	0.057	0.610
Unilateral	16 (22.9)	15 (27.8)	3 (18.8)					
Bilateral	4 (5.7)	16 (29.6)	4 (25.0)					
Total	20 (28.6)	31 (57.4)	7 (43.8)	0.005	0.402	0.002	0.378	0.498

Abbreviations: ABI, ankle-brachial index; ASCVD, atherosclerotic cardiovascular disease; baPWV, brachial-ankle pulse wave velocity; BMI, body mass index; BRVO, branch retinal vein occlusion; CRVO, central retinal vein occlusion; eGFR, estimated glomerular filtration rate; HbA1c, hemoglobin A1C; HDL-C, high-density lipoprotein cholesterol; IMT, intima-media thickness; LDL-C, low-density lipoprotein cholesterol; RVO, retinal vein occlusion; SMD, standardized mean difference.

Supplementary Table S2. Statistical powers of binomial logistic regression analyses for the predictors of RVO

				Univariate				Multivariate				
		RVO (-)	RVO (+)	OR	95% CI	*p*-value	*Statistical power*	OR	95% CI	*p*-value	*Partial R^2**	*Statistical power*
Smoking	No	55	48	1.68	0.78 - 3.60	0.182	0.259	2.86	1.16 - 7.04	0.022	0.038	0.714
	Yes	15	22									
LDL-C (per 30 mg/dL)		70	70	1.54	1.06 - 2.24	0.024	0.724	1.60	1.05 - 2.44	0.03	0.038	0.776
baPWV (per 5 m/s)		70	70	2.25	1.25 - 4.04	0.007	0.998	2.00	1.03 - 3.89	0.041	0.03	0.981
Carotid plaque	No	50	32	2.97	1.47 - 5.98	0.002	0.854	3.15	1.38 - 7.16	0.006	0.056	0.865
	Yes	20	38									

*Partial R^2 of the independent variable of interest with other independent variables in the model.

Abbreviations: baPWV, brachial-ankle pulse wave velocity; CI, confidence interval; LDL-C, low-density lipoprotein cholesterol; OR, odds ratio; RVO, retinal vein occlusion.

Supplementary Table S3. Statistical powers of multinomial logistic regression analyses for the predictors of RVO

	BRVO vs. Control			CRVO vs. Control			BRVO vs. Control				CRVO vs. Control			
	OR	*p*	*statistical power*	OR	*p*	*statistical power*	OR	*p*	*Partial R^2*	*statistical power*	OR	*p*	*Partial R^2*	*statistical power*
Smoking	1.41 (0.62-3.22)	0.414	0.127	2.85 (0.91-8.92)	0.072	0.506	2.41 (0.91-6.34)	0.075	0.025	0.523	4.58 (1.26-16.6)	0.021	0.067	0.801
LDL-C (per 30 mg/dL)	2.17 (1.39-3.38)	0.001	0.99	0.58 (0.30-1.12)	0.103	0.502	2.31 (1.42-3.75)	0.001	0.088	0.993	0.61 (0.31-1.21)	0.156	0.023	0.421
baPWV (per 5 m/s)	2.16 (1.17-4.00)	0.014	0.989	2.56 (1.09-6.03)	0.032	0.924	1.83 (0.92-3.66)	0.086	0.021	0.909	2.54 (0.98-6.59)	0.056	0.064	0.9
Carotid plaque	3.37 (1.59-7.12)	0.001	0.899	1.94 (0.64-5.93)	0.243	0.251	3.94 (1.65-9.41)	0.002	0.025	0.937	2.01 (0.54-7.50)	0.300	0.023	0.269

*Partial R^2 of the independent variable of interest with other independent variables in the model.

Abbreviations: baPWV, brachial-ankle pulse wave velocity, BRVO, branch retinal vein occlusion; CRVO, central retinal vein occlusion; LDL-C, low-density lipoprotein cholesterol; OR, odds ratio; RVO, retinal vein occlusion.

Supplementary Figure S1. Efficacy of the propensity score matching

Age and sex were matched between the patients with RVO and those without RVO using the propensity scores, and the histograms and the absolute mean difference showed that age and sex were well balanced after the matching process.

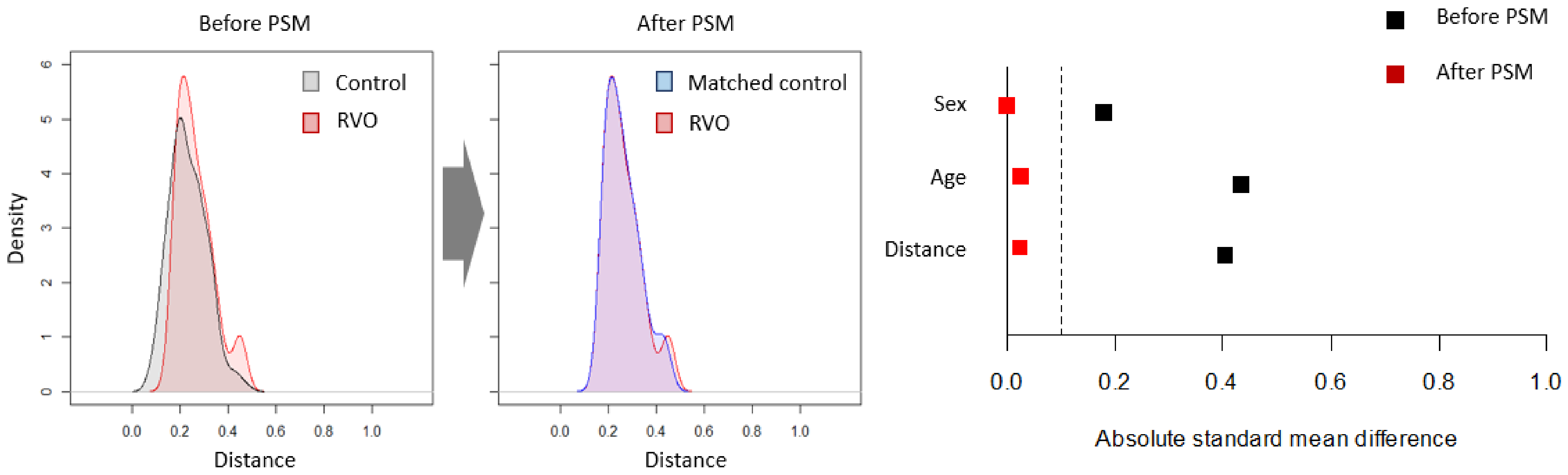

PSM, propensity score matching; RVO, retinal vein occlusion
